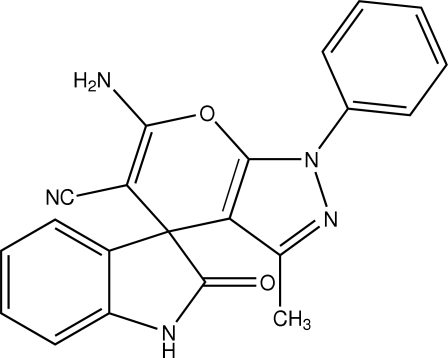# 6′-Amino-3′-methyl-2-oxo-1′-phenyl-1′,3a′,4′,7a′-tetra­hydrospiro­[1*H*-indole-3(2*H*),4′-pyrano[2,3-*d*]pyrazole]-5′-carbonitrile. Corrigendum

**DOI:** 10.1107/S1600536808005564

**Published:** 2008-03-05

**Authors:** S. Etti, G. Shanthi, G. Shanmugam, P. T. Perumal

**Affiliations:** a Centre of Advanced Study in Crystallography and Biophysics, University of Madras, Guindy Campus, Chennai 600 025, India; b Organic Chemistry Division, Central Leather Research Institute, Adyar, Chennai 600 020, India

## Abstract

The title and chemical structural diagram in Etti, Shanmugam & Perumal [*Acta Cryst.* (2008), E**64**, o341] are corrected.

## Abstract 2

Corrigendum to *Acta Cryst.* (2008), E**64**, o341.

In the paper by Etti, Shanmugam & Perumal [*Acta Cryst.* (2008), E**64**, o341], the chemical name in the title and the structual diagram are incorrect. The correct title should be ‘6′-Amino-3′-methyl-2-oxo-1′-phenyl-1′,4′-tetra­hydrospiro­[1*H*-indole-3(2*H*),4′-pyrano[2,3-*d*]pyrazole]-5′-carbonitrile’ and the correct scheme is shown below.